# Fatty Acids and Protein Content of Underexplored Tropical Palm Fruits

**DOI:** 10.1007/s11130-026-01521-0

**Published:** 2026-05-15

**Authors:** Salima Haddou, Mohamed Ezzaitouni, Tarik Chileh-Chelh, Ana Minerva García-Cervantes, Miguel Ángel Rincón-Cervera, Ferdaous Al-Ferjani, Ignacio Manuel Rodríguez-García, Chahine Abdelkrim, José Luis Guil-Guerrero

**Affiliations:** 1 Department of Agronomy, Food Technology Area, Escuela Politécnica Superior, Universidad de Almería, 04120 Almería, Spain; 2 Laboratory of Advanced Materials and Process Engineering, Faculty of Science, University Ibn Tofail, BP 242, Kenitra, Morocco; 3 Department of Chemistry and Physics, Faculty of Experimental Sciences, CIAMBITAL. Universidad de Almería, 04120 Almería, Spain

**Keywords:** Seed oil, Fruit pulp, Stearoyl-CoA desaturase, Nutritional indices, Fatty acid profiles

## Abstract

The Arecaceae family encompasses numerous tropical palms with notable nutritional and bioactive potential; however, tropical palm fruit species remain comparatively underexplored. This study characterised the biochemical composition of fruits from eight species: *Attalea phalerata*, *Bactris gasipaes*, *Copernicia alba*, *Latania verschaffeltii*, *Syagrus kellyana*, *Syagrus orinocensis*, *Veitchia metiti*, and *Wodyetia bifurcata*. In fruit pulps, substantial interspecific variability was observed in moisture (8.5–87.7%), total fatty acids (1.3–4.7% of dry weight), and protein contents (5.2–7.3% of dry weight). Fatty acid profiles of fruit pulps differed markedly in the proportions of polyunsaturated (PUFA, 12.0–41.8%), monounsaturated (MUFA, 16.3–40.5%), and saturated fatty acids (SFA, 34.2–47.6%), resulting in distinct nutritional indices among species, including atherogenic (AI, 0.3–1.5), thrombogenic (TI, 0.3–0.7), hypocholesterolemic/hypercholesterolemic (HH, 0.4–2.5), and stearoyl-CoA desaturase (SCD) indices. The fruit pulps of *B. gasipaes* and *L. verschaffeltii* exhibited particularly favourable lipid profiles, with n-6/n-3 ratios below 1.0, indicating potential cardiovascular benefits. Seeds of *W. bifurcata* exhibited the highest HH index (2.3), indicating a pronounced hypocholesterolemic potential. Overall, these results demonstrate considerable biochemical diversity among tropical palm fruit and underscore their potential as alternative sources of lipids and plant proteins, supporting their potential use as human food ingredients.

**Supplementary Information:**

The online version contains supplementary material available at 10.1007/s11130-026-01521-0.

## Introduction

The Arecaceae family is native to tropical and subtropical regions worldwide and is among the largest and most economically important plant families. Species within this family provide diverse resources, including wood, fibres, oils, waxes, food products, and beverages that substantially support rural livelihoods and contribute to international trade [1]. Among these resources, palm fruits and seeds are particularly valued for their lipid content, with fatty acids (FAs) constituting key bioactive and industrially relevant components. Despite their importance, the nutritional and functional diversity of many tropical palm species, especially those underexplored for date production, remains insufficiently characterised [2]. In many tropical regions, Arecaceae fruits and seeds have long been integrated into traditional diets and local food systems, frequently serving as sources of edible oils [3]. Some studies suggest a potential associations between the consumption of certain palm fruits and improvements in lipid metabolism and antioxidant status [4].

Oils from palm pulp and seeds typically contain complex mixtures of saturated FAs (SFA), monounsaturated FAs (MUFAs), and polyunsaturated FAs (PUFAs), with proportions that vary widely among species and between pulp and seed tissues. This compositional variability directly influences nutritional quality, oxidative stability, and technological functionality [2]. A key nutritional indicator in lipid evaluation is the *n*−6/*n*−3 PUFA ratio. Ratios below 4:1 are often considered cardioprotective and anti-inflammatory, whereas excessive *n*−6 intake relative to *n*−3 has been associated with pro-inflammatory and atherogenic effects [5]. In many palm species, oleic acid (OA, C18:1*n*−9) and palmitic acid (PA, C16:0) predominate [6], while lauric acid (LaA, C12:0), myristic acid (MA, C14:0), linoleic acid (LA, C18:2*n*−6), and α-linolenic acid (ALA, C18:3*n*−3) occur in variable proportions depending on species, maturity stage, and environmental conditions [7, 8]. These compositional differences largely determine whether an oil is better suited for nutritional, cosmetic, or industrial applications. Medium-chain SFAs (MCSFAs) are rapidly oxidised for energy, whereas long-chain SFAs (LCSFAs) have been associated with increased cardiovascular risk [7]. Although the overall health impact of SFAs remains debated, emerging evidence increasingly distinguishes between MCSFAs and LCSFAs, suggesting that MCSFAs may exert comparatively more favourable metabolic effects [7]. In contrast, MUFAs, particularly OA, have been associated with anti-atherogenic, anti-inflammatory, and insulin-sensitising properties [9]. Likewise, ALA, the principal plant-derived *n*−3 PUFA, plays an essential role in maintaining membrane fluidity and supporting cardiovascular health. Consequently, palm oils containing balanced proportions of MUFA and PUFA with favourable *n*−6/*n*−3 ratios are of particular nutritional interest [8].

Despite expanding research on economically important palms, the FA composition of tropical date palms and other underexplored Arecaceae species remains poorly documented. For example, *Bactris gasipaes* seed oil is characterised by high levels of MCFAs [3]. In contrast, the seed oils of *Archontophoenix cunninghamiana* and *Trachycarpus fortunei* contain elevated proportions of LCPUFAs, such as LA [10]. Other species within the family display lipid profiles dominated by LCSFAs, potentially limiting their nutritional appeal while enhancing their industrial suitability [2]. Collectively, these findings underscore the substantial biochemical heterogeneity within the Arecaceae family.

On this basis, the present study hypothesises that the pulp and seeds of tropical fruits of the Arecaceae family have distinct lipid and protein profiles that could allow the identification of species with a higher nutritional or functional value under the environmental conditions of the Canary Islands. Specifically, this work aims to characterise and compare the FA composition, lipid nutritional quality indices, protein content, and moisture levels of the pulp and seed from fruits of eight tropical Arecaceae species.

## Materials and Methods

The Materials and Methods section is presented as supplementary material (Supplementary Material S1).

### Samples

**Fig. 1:**
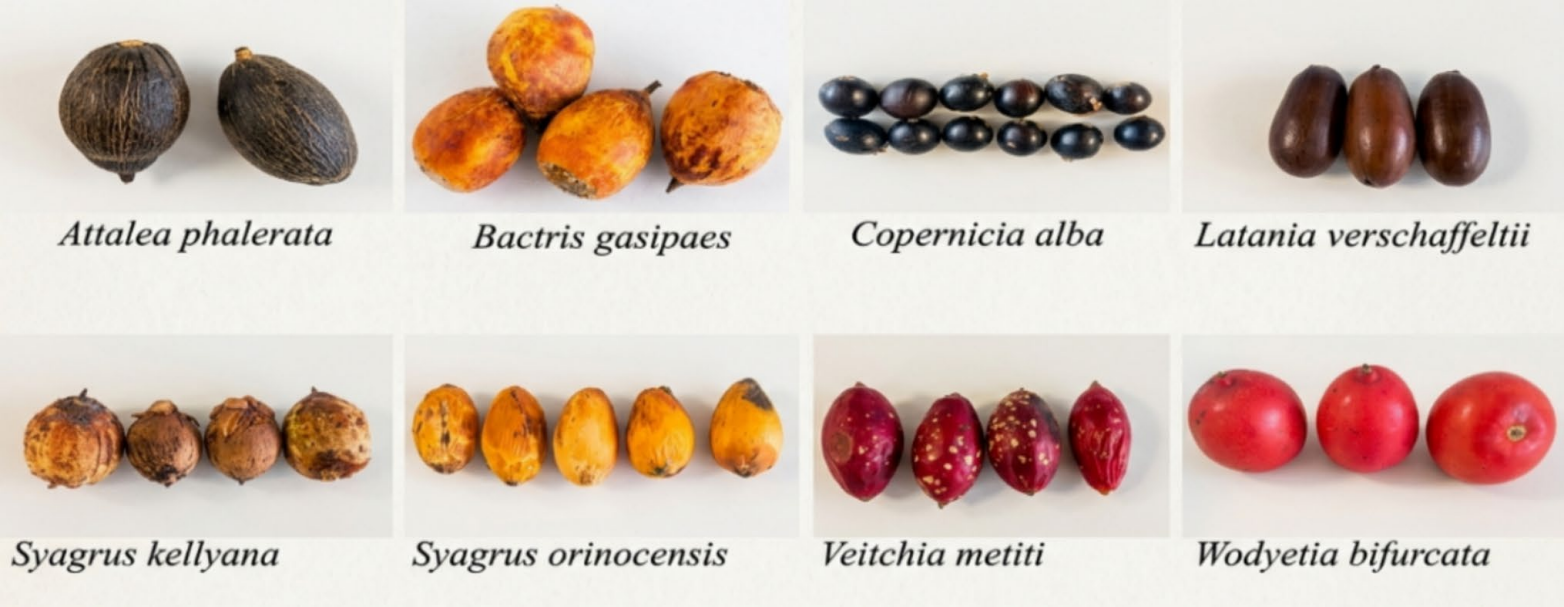
Fruits of the species analysed in this study

Eight selected date species were collected in October 2024 at the *Palmetum garden* (Geographic coordinates: 28.452334, −16.2563; Santa Cruz de Tenerife, Canary Islands, Spain). This area is characterized by a subtropical coastal climate, with mild temperatures throughout the year, low annual rainfall, and high solar radiation.All tropical palm fruits included in this study (Fig. 1) are edible species (Supplementary Table S2), and details on common names and species ranges are provided in the Supplementary Table S3.

## Results and Discussion

### Moisture Content

Moisture content values are presented in Table 1. In all species analysed, seeds exhibited substantially lower moisture levels than the corresponding pulps. Seed (endosperm) moisture ranged from 3.6 g/100 g in *A. phalerata* to 12.0 g/100 g in *W. bifurcata*, while *B. gasipaes*, *L. verschaffeltii*, *S. orinocensis*, *V. metiti*, and *C. alba* seeds showed intermediate values (4.6–9.1 g/100 g). No seeds were recovered from *S. kellyana*. Marked interspecific variation was observed in fruit pulps moisture content. *V. metiti* presented the highest value (87.7 g/100 g), whereas *C. alba* exhibited a markedly lower level (8.5 g/100 g). Such differences are technologically and nutritionally relevant, as moisture strongly influences texture, freshness, microbial stability, and shelf life [11]. Given that all the fruits were harvested ripe in the same place, the differences in moisture content would be attributable to genetic factors. High-moisture fruit pulp species, such as *V. metiti*, *S. kellyana*, *L. verschaffeltii*, and *W. bifurcata* are likely to be more susceptible to microbial spoilage and postharvest deterioration, particularly under humid tropical conditions. Conversely, the low moisture observed in fruit pulps of *C. alba* and, to a lesser extent, in *A. phalerata* and *B. gasipaes*, suggests greater storage stability due to reduced water activity, which limits microbial growth and metabolic processes [12]. Compared with Middle Eastern date cultivars, which typically undergo dehydration during ripening and contain lower water levels at full maturity, the pulp of some tropical palm fruits appear relatively more perishable. This characteristic may impose additional postharvest management challenges, especially in regions lacking controlled storage infrastructure [12]. The relationship between high moisture and accelerated deterioration is consistent with general patterns reported for Arecaceae species and other tropical fruits [13, 14]. However, because this study did not include long-term storage trials or controlled inoculation assays, the precise implications for decay kinetics and seed viability remain unclear. Overall, the substantial variability in moisture content among species highlights the importance of species-specific handling, preservation strategies, and potential processing approaches for these underexplored tropical palms.

**Table 1:** Moisture and protein of the pulp and seeds of Arecaceae fruits examined in this study ^a,b,c^

Code	Species	Pulp		Seeds (endosperm)	
		Moisture g/100 g	Protein g/100 g dw	Moisture g/100 g	Protein g/100 g dw
AP	*Attalea phalerata*	43.1 ± 0.6^d^	6.3 ± 0.0^ cd^	3.6 ± 0.2^ g^	15.3 ± 0.3^a^
BG	*Bactris gasipaes*	52.9 ± 1.5^c^	7.3 ± 0.0^a^	4.6 ± 0.3^f^	7.4 ± 0.0^b^
CA	*Copernicia alba*	8.5 ± 0.1^e^	5.2 ± 0.0^e^	8.5 ± 0.1^e^	6.2 ± 0.0^e^
LV	*Latania verschaffeltii*	60.5 ± 3.1^bc^	5.2 ± 0.0^e^	9.1 ± 0.0^b^	7.5 ± 0.0^b^
SK	*Syagrus kellyana*	58.8 ± 1.8^bc^	6.9 ± 0.0^b^	-	-
SO	*Syagrus orinocensis*	55.2 ± 2.5^c^	6.2 ± 0.0^d^	7.0 ± 0.3^d^	7.1 ± 0.0^c^
VM	*Veitchia metiti*	87.7 ± 8.4^a^	6.5 ± 0.1^c^	8.5 ± 0.3^c^	6.7 ± 0.0^d^
WB	*Wodyetia bifurcata*	66.7 ± 1.7^b^	6.4 ± 0.2^ cd^	12.0 ± 0.2^a^	4.7 ± 0.0^f^

^a^ Data represent mean ± standard deviation of three samples analysed in triplicate each. ^b^Differences in moisture and protein content of the various samples were tested according to one-way ANOVA followed by Tukey’s post hoc test. ^c^Within a column, means followed by different letters are significantly different at *p* < 0.05. (-) Absence of seed

### Protein Content

#### Protein Content of Fruit Pulps

Protein concentrations in the fruit pulp ranged from 5.2 to 7.3 g/100 g dw (Table 1), indicating moderate protein levels compared with most fleshy fruits. The highest value was recorded in *B. gasipaes* (7.3 g/100 g dw), followed by *S. kellyana* (6.9 g/100 g dw) and *V. metiti* (6.5 g/100 g dw). These differences may reflect genetic background and/or fruit maturity at harvest [15], since all fruits were collected at the same environmental conditions. Overall, the values obtained are consistent with previous reports for Arecaceae fruits. For example, *Mauritia flexuosa* has been reported to contain 5.9 g/100 g dw of protein [16], which is comparable to the protein content measured in the fruit pulp of *Attalea phalerata*, *Veitchia* metiti, and *W. bifurcata* (6.3–6.5 g/100 g dw). In addition, earlier studies reported protein contents of 6.8–7.5 g/100 g dw in *B. gasipaes* [15], corroborating the present findings. Although these fruits do not reach the protein concentrations typical of tree nuts, their levels are notable within the fruit category and support the potential of *B. gasipaes* as a complementary plant-based protein source [15]. Nevertheless, further research is required to assess protein digestibility and amino acid composition.

#### Protein Content of Seeds

Seed protein content showed greater interspecific variation, ranging from 4.7 to 15.3 g/100 g dw (Table 1). *A. phalerata* exhibited the highest value (15.3 g/100 g dw), whereas *W. bifurcata* showed the lowest (4.7 g/100 g dw). Most other species fell within 6.0–7.5 g/100 g dw, consistent with typical palm seed values. The protein level in *A. phalerata* markedly exceeds previously reported values for *B. gasipaes* (3.3–4.6 g/100 g dw) and other Arecaceae seeds, including *Astrocaryum vulgare* (3.0–4.6 g/100 g dw) [17]. This discrepancy may be due to geographical and genetic factors, as well as differences in analytical procedures for protein quantification and sample preparation. *B. gasipaes* has been described as a low-protein seed [15], consistent with our results. Seeds containing 6–7% protein remain nutritionally relevant but may be less suitable for high-protein formulations without further processing [18].

### Fatty Acid Profiles

#### Fatty Acid Profiles of Fruit Pulp Oils

As shown in Table 2, total FA content in the fruit pulps varied markedly among species, ranging from 1.3 in *W. bifurcata* to 4.7 g/100 g dw in *B. gasipaes*. These relatively high lipid levels distinguish the analysed fruit pulps from conventional sweet dates and position them closer to oil-bearing fruits in terms of composition [8]. The highest proportion of SFAs was recorded in *C. alba* (47.6%), whereas *B. gasipaes* and *V. metiti* showed the lowest values (~ 34%). PA was the predominant SFA in all species, ranging from 13.6% in *C. alba* to 33.4% in *S. kellyana*, while *A. phalerata*, *L. verschaffeltii*, *S. orinocensis*, and *V. metiti* exhibited intermediate levels (27–32%). Regarding MCFAs, *C. alba* was distinguished by comparatively high CA (4.8%), LaA (11.9%), and MA (13.5%) contents. Owing to its CA and LaA levels, this species may represent a candidate for functional oil development. In contrast, these FAs were absent or present only in minor amounts in most of the other analysed taxa [19].

**Table 2:** Fatty acid profiles of the fruit pulp of Arecaceae species studied here compared with those of common Arecaceae species^a,b,c^

Fatty acids	Species/Code									
	*A. phalerata*	*B. gasipaes*	*C. alba*	*L. verschaffeltii*	*S. kellyana*	*S. orinocensis*	*V. metiti*	*W. bifurcata*	*P. dactylifera* var	*P. dactylifera* var *Medjool*
	AP	BG	CA	LV	SK	SO	VM	WB	*Deglet Nour*[24]	[24]
FAs (g/100 g DW)	2.1 ± 0.3^e^	4.7 ± 0.2^a^	3.1 ± 0.2^d^	1.6 ± 0.1^f^	3.7 ± 0.1^c^	4.1 ± 0.2^b^	3.5 ± 0.2^c^	1.3 ± 1.2^f^	0.1 ± 0.1^ g^	0.2 ± 0.2^ g^
∑ SFA	41.9 ± 1.6^c^	34.2 ± 0.3^e^	47.6 ± 5.1^b^	37.7 ± 0.7^d^	41.0 ± 0.1^c^	42.1 ± 0.6^c^	34.7 ± 1.2^e^	35.3 ± 0.2^de^	46.3 ± 2.2^b^	48.4 ± 2.8^a^
8:0 (CA)	< LOQ	< LOQ	4.8 ± 0.5^a^	< LOQ	< LOQ	< LOQ	< LOQ	< LOQ	n.r	1.0 ± 0.5^b^
10:0	< LOQ	< LOQ	< LOQ	< LOQ	< LOQ	< LOQ	< LOQ	< LOQ	n.r	1.1 ± 0.5^a^
11:0 (UA)	1.0 ± 0.1^a^	< LOQ	< LOQ	0.7 ± 0.0^b^	0.4 ± 0.1^ cd^	0.7 ± 0.1^bc^	0.1 ± 0.1^de^	0.5 ± 0.2^bc^	n.r	n.r
12:0 (LaA)	0.7 ± 0.0^d^	0.6 ± 0.2^d^	11.9 ± 0.6^b^	0.3 ± 0.1^d^	0.7 ± 0.0^d^	0.7 ± 0.1^d^	0.3 ± 0.2^d^	0.3 ± 0.0^d^	13.0 ± 0.7^a^	10.3 ± 0.7^c^
14:0 (MA)	0.5 ± 0.4^d^	0.6 ± 0.0^d^	13.5 ± 1.5^a^	0.3 ± 0.1^d^	1.1 ± 0.0^d^	1.3 ± 0.3^d^	0.2 ± 0.0^d^	0.3 ± 0.1^d^	5.5 ± 0.4^c^	9.5 ± 0.2^b^
15:0 (PEA)	0.6 ± 0.0^bcd^	0.8 ± 0.1^a^	0.4 ± 0.1^d^	0.4 ± 0.1^ cd^	0.6 ± 0.2^abc^	0.8 ± 0.1^ab^	< LOQ	0.6 ± 0.1^bcd^	n.r	n.r
16:0 (PA)	31.3 ± 1.1^b^	26.3 ± 0.1^ef^	13.6 ± 0.9^ g^	27.6 ± 0.5^de^	33.4 ± 0.2^a^	32.9 ± 0.4^a^	29.6 ± 1.1^c^	28.7 ± 0.5^ cd^	25.7 ± 1.0^f^	24.9 ± 0.8^f^
17:0 (MA)	1.2 ± 0.1^a^	0.5 ± 0.0^b^	0.5 ± 0.5^b^	0.5 ± 0.1^b^	0.6 ± 0.0^b^	1.4 ± 0.1^a^	0.6 ± 0.1^b^	0.4 ± 0.0^b^	n.r	n.r
18:0 (SA)	6.2 ± 0.3^a^	4.9 ± 0.0^ab^	2.8 ± 1.0^c^	5.0 ± 1.5^ab^	3.7 ± 0.0^bc^	3.8 ± 0.3^bc^	4.0 ± 0.1^bc^	2.6 ± 0.9^c^	n.r	n.r
20:0 (AA)	0.5 ± 0.3^c^	0.5 ± 0.1^c^	< LOQ	2.8 ± 0.4^a^	0.5 ± 0.1^c^	0.5 ± 0.0^c^	. < LOQ	1.8 ± 0.8^b^	2.1 ± 0.1^b^	1.6 ± 0.1^b^
∑ MUFA	16.3 ± 0.6^f^	31.6 ± 0.5^d^	40.5 ± 0.5^b^	35.6 ± 3.4^c^	17.5 ± 0.0^f^	17.4 ± 0.3^f^	23.1 ± 0.5^e^	36.0 ± 3.3^c^	44.8 ± 0.2^a^	41.9 ± 1.2^b^
16:1*n*−7 (POA)	0.3 ± 0.1^ cd^	1.1 ± 0.1^b^	4.3 ± 0.5^a^	0.5 ± 0.1^bcd^	0.8 ± 0.0^bc^	0.9 ± 0.2^bc^	0.6 ± 0.0^bcd^	0.4 ± 0.1^bcd^	n.r	n.r
18:1*n*−9 *cis* (OA)	10.9 ± 0.7^f^	26.5 ± 0.1^c^	20.1 ± 0.4^d^	29.8 ± 3.5^b^	9.6 ± 0.2^f^	10.0 ± 0.9^f^	16.1 ± 2.0^e^	30.4 ± 0.7^b^	44.8 ± 0.2^a^	41.9 ± 1.2^a^
18:1*n*−7	0.2 ± 0.1^c^	0.6 ± 0.0^bc^	1.6 ± 0.1^ab^	1.7 ± 0.8^a^	< LOQ	0.4 ± 0.1^c^	0.8 ± 0.0^bc^	2.1 ± 0.5^a^	n.r	n.r
20:1*n*−9 (GOA)	0.8 ± 0.3^a^	0.5 ± 0.1^ab^	0.8 ± 0.3^a^	0.6 ± 0.4^a^	0.5 ± 0.2^ab^	0.6 ± 0.0^ab^	0.5 ± 0.1^ab^	0.6 ± 0.5^a^	n.r	n.d
22:1*n*−9 (EA)	3.5 ± 0.4^bcd^	2.5 ± 0.4^ cd^	12.9 ± 2.0^a^	2.3 ± 0.0^d^	4.9 ± 0.1^b^	4.3 ± 0.9^bc^	4.5 ± 1.3^b^	1.8 ± 0.8^de^	n.r	n.r
24:1*n*−9	0.6 ± 0.1^ cd^	0.4 ± 0.0^ cd^	0.7 ± 0.1^bc^	0.6 ± 0.2^ cd^	1.7 ± 0.1^a^	1.3 ± 0.5^ab^	0.8 ± 0.4^bc^	0.8 ± 0.0^bc^	n.r	n.r
∑ PUFA	41.8 ± 0.9^a^	34.1 ± 0.2^b^	12.0 ± 0.4^de^	26.7 ± 2.7^c^	41.6 ± 0.0^a^	40.4 ± 0.1^a^	42.2 ± 1.7^a^	28.7 ± 2.3^c^	10.2 ± 2.6^e^	15.6 ± 3.2^d^
18:2*n*−6 *cis* (LA)	31.0 ± 0.8^ab^	14.3 ± 0.2^c^	5.9 ± 0.6^d^	11.5 ± 2.0^c^	27.5 ± 0.3^b^	27.8 ± 0.0^b^	32.7 ± 2.2^a^	14.1 ± 2.9^c^	5.1 ± 1.3^d^	7.8 ± 1.6^d^
16:3*n*−3	0.6 ± 0.3^b^	0.8 ± 0.3^b^	< LOQ	. < LOQ	1.5 ± 0.2^a^	0.6 ± 0.1^b^	. < LOQ	. < LOQ	n.r	n.r
18:3*n*−3 (ALA)	10.2 ± 1.4^d^	19.1 ± 0.1^a^	6.1 ± 0.2^e^	15.1 ± 0.6^b^	12.3 ± 0.1^bcd^	12.1 ± 0.0^ cd^	9.5 ± 3.8^d^	14.6 ± 0.5^bc^	4.2 ± 0.1^ef^	2.4 ± 0.2^f^
*∑ n*−6 PUFA	31.0 ± 0.8^ab^	14.3 ± 0.2^c^	6.1 ± 0.2^d^	11.1 ± 2.0^c^	27.8 ± 0.2^b^	27.8 ± 0.0^b^	32.7 ± 2.2^a^	14.1 ± 2.9^c^	5.1 ± 1.3^d^	7.8 ± 1.6^d^
*∑ n*−3 PUFA	10.8 ± 1.7^cde^	19.9 ± 0.4^a^	5.9 ± 0.6^f^	15.1 ± 0.6^b^	13.8 ± 0.3^bc^	12.7 ± 0.1^bcd^	9.5 ± 3.8^de^	14.6 ± 0.6^b^	5.1 ± 1.0^f^	7.8 ± 1.5^ef^
*n*−6/*n*−3	2.9 ± 0.5	0.7 ± 0.0	1.1 ± 0.3	0.8 ± 0.1	2.0 ± 0.1	2.2 ± 0.0	3.8 ± 1.8	1.0 ± 0.2	1.0 ± 0.0	1.0 ± 0.0

^a^ Data represent means ± standard deviation of three samples analyzed in triplicate each. ^b^ Differences in FA amounts/percentages were tested according to one-way ANOVA followed by Tukey’s post hoc test. ^c^ In a row, means followed by different letters are significantly different at *p* < 0.05; Abbreviations: *LOQ* limit of quantitation; *n.r.* not reported; *LaA* lauric acid; *MA* Myristic acid; *PA* palmitic acid; *SA* stearic acid; *POA* palmitoleic acid; *OA* oleic acid; *GOA* eicosenoic acid; *LA* linoleic acid; *ALA* α-linolenic acid; *Total FAs* total fatty acids; *SFA* saturated fatty acid; *MUFA* monounsaturated fatty acid; *PUFA* polyunsaturated fatty acid

MUFA ranged from 16.3% in *A. phalerata* to 40.5% in *C. alba*, with OA as the principal component. *A. phalerata*, *S. kellyana*, and *S. orinocensis* exhibited the lowest MUFA proportions (< 18%), while *W. bifurcata* and *L. verschaffeltii* contained approximately 30% OA. OA, a characteristic FA of olive oil, has been widely investigated for its ability to reduce plasma low-density lipoprotein (LDL) without adversely affecting high-density lipoprotein (HDL) concentrations [20]. Although such effects are primarily documented for olive oil, the presence of substantial OA levels suggests that certain Arecaceae fruits could serve as alternative MUFA sources [2]. PUFAs were highest in *A. phalerata*, *S. kellyana*, *S. orinocensis*, and *V. m*e*titi* (~ 40–42%), largely driven by LA. In contrast, *C. alba* displayed lower total PUFA (< 16%). *B. gasipaes* and *L. verschaffeltii* were notable for elevated ALA contents (19.1 and 15.1%, respectively). The coexistence of LA and ALA in *S. orinocensis* and *V. metiti* is reported here for the first time and is nutritionally relevant, as these essential FAs serve as precursors to long-chain PUFA and have been linked to modulation of inflammatory and metabolic processes [21]. However, these associations indicate functional potential rather than direct clinical outcomes. The relatively high ALA content of *B. gasipaes* is particularly noteworthy, given its role as a precursor of EPA and DHA, compounds associated with anti-inflammatory and neuroprotective effects [22]. The *n*−6/*n*−3 ratio ranged from 0.7 in *B. gasipaes* to 3.8 in *V. metiti*, with *L. verschaffeltii* (0.8) and *W. bifurcata* (1.0) also exhibiting favourable values. Ratios below 4:1 have been associated with reduced risk of inflammatory and cardiovascular disorders, and values below 1 are generally considered particularly advantageous [5]. These findings suggest that the fruit pulps of certain tropical palms combine balanced lipid profiles with appreciable energy density, comparable to oil-bearing fruits such as avocado [23]. In contrast, *P. dactylifera* cultivars (*Deglet Nour* and *Medjool*, Table 2) contain less than 0.2 g/100 g dw of total FAs [24], reflecting their carbohydrate-dominant composition. Thus, the tropical Arecaceae examined here display markedly higher total FA contents, aligning them more closely with oilseed-type fruits than with conventional sweet dates [24]. Moreover, several tropical species showed lower SFA and higher PUFA proportions than *P. dactylifera*, particularly *A. phalerata*, *S. kellyana*, and *V. metiti*, suggesting comparatively more balanced lipid compositions. This study represents the first detailed report of FA profiles in the pulp fruit of these palm species. The elevated lipid concentrations and marked interspecific variability observed highlight their potential as alternative sources of functional lipids for food and nutraceutical applications. Nevertheless, confirmation through multi-site, multi-season, and clinical investigations is required before definitive health-related conclusions can be drawn.

#### Fatty Acid Profiles of Palm Seed Oils

The FA composition in seed oil further reflects the marked diversity within the studied species fruit(Table 3). Total FAs content varied widely, ranging from 0.6 in *W. bifurcata* to 28.8 g/100 g dw in *S. orinocensis*, indicating interspecific differences in energy density and potential applications. SFAs predominated in *B. gasipaes* (87.8%) and *S. orinocensis* (86.5%), whereas *C. alba* and *W. bifurcata* exhibited comparatively lower proportions. MCFA characterised high-SFA species: LaA exceeded 50% in *B. gasipaes* and *S. orinocensis*. MUFA were dominated by OA, reaching 43.2% in *W. bifurcata* and 32.2% in *C. alba*. The OA-rich profiles of *C. alba* and *W. bifurcata* resemble high-quality edible oils; however, compositional similarity should not be interpreted as direct nutritional or cardiovascular equivalence [25]. Similar patterns were reported for *Oenocarpus bataua*, another species recognised as a natural OA source [26]. PUFAs were less abundant overall but remained significant in *W. bifurcata* (26.5%) and *V. metiti* (17.5%). ALA was detected only in trace amounts, limiting the contribution of *n*−3 PUFAs and leading to high *n*−6/*n*−3 ratios. The variability observed from highly SFA-dominant profiles to MUFA-rich compositions is consistent with patterns reported for palm oils [27]. Species like *B. gasipaes*, where LaA exceeds 50%, exhibit profiles similar to coconut and palm kernel seed, traditionally valued in cosmetics for their oxidative stability [28]. In contrast, *W. bifurcata* stands out for higher LA proportions; however, elevated *n*−6/*n*−3 ratios warrant cautious interpretation of functional claims [29]. Seed oils of tropical palm species contain substantially higher total FA levels (most species contain ~ 10–30 g/100 g dw) than seeds of *P. dactylifera* cultivars (Table 3), in which total FAs ranges from 3.4 to 6.4 g/100 g dw [10]. Moreover, species such as *W. bifurcata*, *L. verschaffeltii*, and *C. alba* were particularly rich in MUFA, which are valued for their thermal stability in food processing and seed oil production [30]. The seeds analysed here exhibited broader lipid diversity across SFA, MUFA, and PUFA fractions than others seeds from MUFA-rich *P. dactylifera* cultivars This highlight both nutritional and industrial potential, although further in vivo studies are required to evaluate their physiological and health-related effects.

**Table 3:** Fatty acid profiles of the seed (endosperm) oils of the Arecaceae species studied here, compared with those of common Arecaceae species ^a,b,c^

Fatty acids	Species/Code								
	*B. gasipaes*	*C. alba*	*L. verschaffeltii*	*A. phalerata*	*S. orinocensis*	*V. metiti*	*W. bifurcata*	*P. dactylifera* var	*P. dactylifera* var
	BGS	CAS	LVS	APS	SOS	VMS	WBS	*Deglet Nour* [10]	*Medjool* [10]
FAs (g/100 g)	23.5 ± 5.5^ab^	16.8 ± 3.1^bc^	11.4 ± 5.1^ cd^	9.5 ± 2.0^de^	28.8 ± 4.1^a^	1.9 ± 0.2f	0.6 ± 0.1f	3.4 ± 0.5^ef^	6.4 ± 1.8^def^
∑ SFA	87.8 ± 1.3^a^	56.9 ± 1.3^e^	79.6 ± 0.7^b^	76.7 ± 1.0^c^	86.8 ± 0.7^a^	60.7 ± 0.2^d^	30.1 ± 1.5^ h^	46.0 ± 1.3^f^	42.1 ± 0.0^ g^
8:0 (CA)	3.8 ± 0.3^c^	0.2 ± 0.1^f^	1.0 ± 0.4^e^	9.8 ± 0.1^a^	7.7 ± 0.1^b^	0.9 ± 0.1^e^	2.7 ± 0.1^d^	0.4 ± 0.0^f^	0.4 ± 0.0^f^
10:0	5.0 ± 0.2^bc^	11.4 ± 1.3^a^	< LOQ	5.6 ± 0.0^b^	4.2 ± 0.0^c^	< LOQ	< LOQ	0.5 ± 0.0^d^	0.4 ± 0.1^d^
12:0 (LaA)	51.6 ± 1.1^a^	22.5 ± 1.4^d^	45.0 ± 2.4^b^	40.5 ± 1.3^c^	50.1 ± 0.7^a^	7.7 ± 0.2^f^	1.1 ± 0.3^ g^	22.0 ± 0.4^d^	17.5 ± 0.1^e^
14:0 (MA)	17.8 ± 1.8^c^	13.1 ± 1.4^d^	23.8 ± 1.3^b^	12.2 ± 0.4^de^	16.9 ± 0.2^c^	27.9 ± 0.2^a^	< LOQ	11.1 ± 1.6^e^	10.9 ± 0.0^e^
16:0 (PA)	5.8 ± 0.7^ g^	7.3 ± 0.6^ef^	7.9 ± 0.3^de^	5.9 ± 0.2^ fg^	5.9 ± 0.2^ fg^	20.2 ± 0.0^b^	21.8 ± 1.6^a^	9.7 ± 1.3^ cd^	10.5 ± 0.0^c^
18:0 (SA)	3.8 ± 1.9^abc^	2.5 ± 0.5^bcd^	1.9 ± 0.2^d^	2.7 ± 0.1^bcd^	2.1 ± 0.2^d^	4.1 ± 0.6^ab^	4.6 ± 0.1^a^	3.2 ± 0.2^abcd^	2.4 ± 0.6^ cd^
∑ MUFA	9.2 ± 0.9^ g^	36.2 ± 0.4^c^	15.4 ± 0.2^f^	18.0 ± 0.7^e^	9.4 ± 0.3^ g^	21.8 ± 0.7^d^	43.4 ± 0.7^a^	41.8 ± 1.1^b^	44.1 ± 0.9^a^
16:1*n*−7 (POA)	< LOQ	4.0 ± 1.0^a^	< LOQ	0.2 ± 0.0^b^	< LOQ	< LOQ	< LOQ	n.r	n.r
18:1*n*−9 *cis* (OA)	9.2 ± 0.9^ g^	32.2 ± 0.6^c^	15.4 ± 0.2^f^	17.8 ± 0.7^e^	9.4 ± 0.3^ g^	21.8 ± 0.7^d^	43.4 ± 0.7^ab^	42.5 ± 1.1^b^	44.7 ± 0.2^a^
∑ PUFA	3.0 ± 0.5^ g^	6.9 ± 1.6^e^	4.9 ± 0.9^f^	5.3 ± 0.3^ef^	3.8 ± 0.4^ fg^	17.5 ± 0.5^b^	26.5 ± 0.8^a^	8.9 ± 0.1^d^	11.1 ± 0.8^c^
18:2*n*−6 *cis* (LA)	3.0 ± 0.5^ g^	6.8 ± 1.6^e^	4.8 ± 0.9^f^	5.1 ± 0.2^f^	3.6 ± 0.3^ fg^	17.5 ± 0.5^b^	26.5 ± 0.8^a^	8.9 ± 0.6^d^	10.5 ± 0.0^c^
18:3*n*−3 (ALA)	< LOQ	0.2 ± 0.1^a^	0.2 ± 0.0^a^	0.2 ± 0.1^a^	0.1 ± 0.1^a^	< LOQ	< LOQ	n.r	n.r
*∑ n*−6 PUFA	3.0 ± 0.5^ g^	6.8 ± 1.6^e^	4.8 ± 0.9^f^	5.1 ± 0.2^f^	3.6 ± 0.3 fg	17.5 ± 0.5^b^	26.5 ± 0.8^a^	8.9 ± 0.1^e^	11.1 ± 0.8^d^
*∑ n*−3 PUFA	< LOQ	0.2 ± 0.1^a^	0.2 ± 0.0^a^	0.2 ± 0.1^a^	0.1 ± 0.0^a^	< LOQ	< LOQ	n.r	n.r
*n*−6/*n*−3	< LOQ	45.4 ± 9.8^a^	31.2 ± 5.0^a^	29.2 ± 11.3^a^	34.1 ± 19.2^a^	< LOQ	< LOQ	n.r	2.5 ± 0.7^b^

^a^ Data represent means ± standard deviation of three samples analyzed in triplicate each. ^b^ Differences in FA amounts/percentages were tested according to one-way ANOVA followed by Tukey’s post hoc test. ^c^ In a row, means followed by different letters are significantly different at *p* < 0.05. Abbreviations: *LOQ* limit of quantitation; *n.r.* not reported; *LaA* lauric acid; *MA* Myristic acid; *PA* palmitic acid; *SA* stearic acid; *POA* palmitoleic acid; *OA* oleic acid; *LA* linoleic acid; *ALA* α-linolenic acid; *Total FAs* total fatty acids; *SFA* saturated fatty acid; *MUFA* monounsaturated fatty acid; *PUFA* polyunsaturated fatty acid

### Stearoyl-CoA Desaturase Activity Indices and Nutritional indices

All calculated indices are reported in Supplementary Table S4. SCD_i_14_ was not detected in any pulp fruit or seed oils samples. In fruit pulp oils, SCDi__16_ ranged from 0.8 in *A. phalerata* to 26.8 in *C. alba*, whereas in seed oils it was detected only in *C. alba*, reaching a markedly high value (35.6). SCDi__18_ values were consistently elevated in both fruit pulp and seed oils, varying from 64.2 in *A. phalerata* pulp oil to 92.9 in *C. alba* seed oil. SCDi__20_ was identified only in fruit pulp oils, ranging from 17.4 in *L. verschaffeltii* to 90.6 in *V. metiti*. The atherogenic index (AI) ranged in fruit pulp oils from 0.3 in *S. kellyana* to 1.5 in *C. alba*, while values from seed oils were considerably higher, peaking at 10.6 in *B. gasipaes*. Thrombogenic index (TI) values were generally low in fruit pulp oils (0.3–0.6) but increased substantially in seed oils, reaching 4.5 in *B. gasipaes*. The hypocholesterolemic/hypercholesterolemic (HH) ratio varied in fruit pulp oils from 0.4 (*C. alba*) to 2.5 (*V. metiti*), whereas most seed oils showed very low values (< 0.5), except *W. bifurcata* (2.3), which warrants analytical verification. Stearoyl-CoA desaturase (SCD) introduces a cis double bond between C9 and C10 of SFAs, generating MUFAs such as OA and POA. Compared with SFAs, MUFAs contribute to membrane fluidity and metabolic regulation [31]. Among desaturation indices, SCDi__16_ and SCDi__18_ reflect the conversion of PA and SA, respectively. The consistently high SCDi__18_ values, particularly in *W. bifurcata* fruit pulp oil (92.5%) and *C. alba* seed oil (92.9%), indicate a substantial capacity for OA synthesis. In contrast, the absence of SCDi__14_ and the limited detection of SCDi__20_ suggest low activity at these positions under the experimental conditions [31, 32]. The wide variation in SCDi__16_ points to species-specific regulation of lipid biosynthesis, potentially influenced by genetic and environmental factors [33]. Most fruit pulp oils exhibited favourable AI (0.3–0.7) and TI (0.3–0.6) values, except *C. alba* (AI = 1.5). Conversely, *B. gasipaes*, *S. orinocensis*, and *L. verschaffeltii* seeds displayed elevated AI and TI values, consistent with their higher SFA proportions. Fruit pulp oil HH ratios (0.4–2.5) were generally favourable, particularly in *V. metiti*, whereas seed oil HH ratios were typically low, excepting *W. bifurcate* (2.3). Overall, the fruit pulp oils of *C. alba*, *V. metiti*, and *L. verschaffeltii* combined high desaturation indices with balanced nutritional profiles. Seed oils, especially that of *B. gasipaes*, showed less favourable lipid indices. These differences likely reflect the differential functionality of the FAs contained in the structures of the fruit, pulp and seeds, although genetic factors are also influential, reinforcing the need for species-level evaluation in functional lipid applications [25]. Overall, wild tropical species displayed broader variability than commercial date cultivars, whose lipid indices were comparatively uniform. Although certain taxa, such as *C. alba,* exhibit promising profiles, confirmation of potential cardiovascular benefits requires further in vivo validation.

## Conclusions

This study underscores the nutritional and functional potential of underutilised tropical Arecaceae fruits sampled at identical environmental conditions in Canary Islands, thus, interspecies variation in lipid and protein composition mostly reflects intrinsic species variability. The FA profiles, characterised by high levels of MUFAs, particularly OA, and PUFAs such as LA and ALA, support the potential suitability of these species for functional food applications where lipid balance and oxidative stability are important. Notably, although *B. gasipaes* and *L. verschaffeltii* exhibited favourable n-6/n-3 ratios, the elevated atherogenic and thrombogenic indices observed in *B. gasipaes* seeds warrant careful consideration of both beneficial and potentially adverse lipid attributes. Overall, *C. alba*, *V. metiti*, and *L. verschaffeltii* may represent promising plant resources for further studies based on their chemical composition, particularly their FA profiles. As this study was limited to compositional analysis, future research should focus on bioaccessibility, safety, biological activity, pulp fruit yields, and nutrient stability during processing. In addition, evaluating consumer acceptance and the feasibility of sustainable large-scale cultivation in both native tropical regions and climatically suitable subtropical environments will be essential for the effective integration of these resources into human diets.

## Supplementary Information

Below is the link to the electronic supplementary material.

Supplementary file1 (DOCX 597 KB)

Supplementary file2 (DOCX 28 KB)

Supplementary file3 (DOCX 20 KB)

Supplementary file4 (DOCX 26 KB)

## Data Availability

The data that supports the findings of this study are available within the article.

## Abbreviations

AI: Atherogenic index
ALA: α-Linolenic acid
ANOVA: Analysis of variance
CA: Capric acid
DW: Dry weight
EA: Erucic acid
FA: Fatty acid
FAME: Fatty acid methyl ester
FID: Flame ionization detector
GC: Gas chromatography
GOA: Gondoic acid
HH: Hypocholesterolemic/Hypercholesterolemic fatty acid ratio
LaA: Lauric acid
LA: Linoleic acid
LCFA: Long-chain fatty acid
LCSFA: Long-chain saturated FA
LOD: Limit of detection
LOQ: Limit of quantification
MA: Myristic acid
MCFA: Medium-chain FA
MCSFA: Medium-chain saturated FA
MUFA: Monounsaturated FA
OA: Oleic acid
PA: Palmitic acid
PEA: Pentadecanoic acid
POA: Palmitoleic acid
PUFA: Polyunsaturated FA
SA: Stearic acid
SCD: Stearoyl-CoA desaturase
SCDi: Stearoyl-CoA desaturase index
SD: Standard deviation
SFA: Saturated FA
TI: Thrombogenic inde

## References

[1] Pinheiro R, Silva M, Costa P et al (2022) Peach palm (*Bactris gasipaes* Kunth) and mammee apple (*Mammea americana* L.) seeds: properties and potential of application in industry. LWT Food Sci Technol 170:114084. 10.1016/j.lwt.2022.114089

[2] Guerin C, Serret J, Montúfar R et al (2020) Palm seed and fruit lipid composition: phylogenetic and ecological perspectives. Ann Bot 125(1):157–172. 10.1093/aob/mcz175 31665224 10.1093/aob/mcz175 PMC7080222

[3] Teixeira L, Gerson EI et al (2022) Emerging lipids from Arecaceae palm fruits in Brazil. Molecules 27(13):4188. 10.3390/molecules27134188 35807433 10.3390/molecules27134188 PMC9268242

[4] Voon PT, Lee ST, Wai Ng TK et al (2019) Intake of palm olein and lipid status in healthy adults: a meta-analysis. Adv Nutr 10(4):647–659. 10.1093/advances/nmy122 31095284 10.1093/advances/nmy122 PMC6628844

[5] Simopoulos AP (2008) The omega-6/omega-3 fatty acid ratio, genetic variation, and cardiovascular disease. Asia Pac J Clin Nutr 17(Suppl 1):131–134 18296320

[6] Guedes AMM, Wilhelm AE, Mouraet JIL et al (2023) Bioactive compounds of fractionated palm oil with a higher content of oleic acid. Rev Bras Frutic 45:e-555. 10.1590/0100-29452023555

[7] St-Onge MP, Jones PJ (2002) Physiological effects of medium-chain triglycerides: potential agents in the prevention of obesity. J Nutr 132(3):329–332. 10.1093/jn/132.3.329 11880549 10.1093/jn/132.3.329

[8] Prathap V, Yadav P, Manorama K et al (2025) Optimizing fatty acid composition and nutritional profile of palm oil-based blends for improved functional properties. J Food Meas Charact 19(4):2861–2878. 10.1007/s11694-025-03152-6

[9] Stefan N, Machicao F, Staiger H et al (2005) Polymorphisms in the gene encoding adiponectin receptor 1 are associated with insulin resistance and high liver fat. Diabetologia 48(11):2282–2291 16205883 10.1007/s00125-005-1948-3

[10] Rincón-Cervera MA, González-Barrio R, Guil-Guerrero JL et al (2023) Arecaceae seeds constitute a healthy source of fatty acids and phenolic compounds. Plants (Basel) 12(2):226. 10.3390/plants12020226 36678939 10.3390/plants12020226 PMC9867020

[11] Lohita B, Srijaya M et al (2024) Novel technologies for shelf-life extension of food products as a competitive advantage: A review. Food Prod Divers Saf Clim Chang:285–306. 10.1007/978-3-031-51647-4_24

[12] Gidado MJ, Gunny AAN, Gopinath CB et al (2024) Challenges of postharvest water loss in fruits: mechanisms, influencing factors, and effective control strategies–a comprehensive review. J Agric Food Res 17:101249. 10.1016/j.jafr.2024.101

[13] Al-Habsi N (2025) Date palm (*Phoenix dactylifera L*.) fruit: strategic crop for food security, nutritional benefits, postharvest quality, and valorization into emerging functional products. Sustainability 17(16):7491. 10.3390/su17167491

[14] Kader AA (2002) Postharvest technology of horticultural crops Ed. 3,vii+-535.

[15] Soares SD, Santos OVD, Nascimento FDCAD et al (2022) A review of the nutritional properties of different varieties and byproducts of peach palm (*Bactris gasipaes*) and their potential as functional foods. Int J Food Prop 25(1):2146–2165. 10.1080/10942912.2022.2127761

[16] Nascimento S, Rodrigues NR, Silva FA et al (2020) Physicochemical composition and antioxidants of buriti (*Mauritia flexuosa Linn. F*.) pulp and sweet. J Bioenergy Food Sci 7(1):e2792019JBFS. 10.18067/jbfs.v7i1.279

[17] Gualberto LS, Ibiapina A, Dias BB et al (2025) Investigation of the physicochemical, bioactive properties and antioxidant potential of seeds of native fruits from Brazil: a study on the tucumã (*Astrocaryum vulgare*), bacupari (*Garcinia gardneriana*) and pupunha (*Bactris gasipaes*). An Acad Bras Cienc 97(1):e20240862. 10.1590/0001-3765202520240862 40136191 10.1590/0001-3765202520240862

[18] Tan M, Nawaz MA, Buckow R (2023) Functional and food application of plant proteins–a review. Food Rev Int 39(5):2428–2456. 10.1080/87559129.2021.1955918

[19] Ramirez A, B JC, C M et al (2026) Chemical composition, nutritional characteristics, and health effects of calafate (*Berberis microphylla*). Plant Foods Hum Nutr 81(1):3. 10.1007/s11130-025-01444-2 10.1007/s11130-025-01444-2 41420747

[20] Farràs M, Canyelles M, Fitó M et al (2020) Effects of virgin olive oil and phenol-enriched virgin olive oils on lipoprotein atherogenicity. Nutrients 12(3):601. 10.3390/nu12030601 32110861 10.3390/nu12030601 PMC7146215

[21] Calder PC (2012) Long-chain fatty acids and inflammation. Proc Nutr Soc 71(2):284–289. 10.1017/S0029665112000067 22369781 10.1017/S0029665112000067

[22] Dimopoulou M, Kolonas A, Stagos D et al (2025) A review of the sustainability, chemical composition, bioactive compounds, antioxidant and antidiabetic activity, neuroprotective properties, and health benefits of microalgae. Biomass Bioenergy 5(1):11. 10.3390/biomass5010011

[23] Kamel BS, Kakuda Y (1994) Tropical fruits: a source of lipids, in Technological Advances in improved and alternative sources of lipids. Springer US, pp. 116–149. 10.1007/978-1-4615-2109-9_5

[24] Lahlou A, Chilh-Chelh T, Lyashenko S et al (2022) Arecaceae fruits: fatty acids, phenolic compounds, and in vitro antitumor activity. Food Biosci 50:102181. 10.1016/j.fbio.2022.102181

[25] Montúfar G, Rommel J (2010) *Oenocarpus bataua* Mart.(Arecaceae): rediscovering a source of high oleic vegetable oil from Amazonia. J Am Oil Chem Soc 87(2):167–172

[26] Kelley DS, Vemuri M, Adkins Y et al (2006) Fatty acid composition of liver, adipose tissue, spleen, and heart of mice fed diets containing t10, c12-, and c9, t11-conjugated linoleic acid. Prostaglandins PLEFA 74(5):331–338. 10.1016/j.plefa.2006.02.008 10.1016/j.plefa.2006.02.008 16631360

[27] Ngando-Ebongue GF, Ajambang WN, Koona P et al (2011) Oil palm, in Technological innovations in major world oil crops. Springer 1: Breeding, pp. 165–200. 10.1007/978-1-4614-0356-2_7

[28] Marina AM, Che Man YB, Nazimah SAH et al (2009) Chemical properties of virgin coconut oil. J Am Oil Chem Soc 86(4):301–307. 10.1007/s11746-009-1351-1

[29] Santos HO, Price JC, Bueno AA (2020) Beyond fish oil supplementation: the effects of alternative plant sources of omega-3 polyunsaturated fatty acids upon lipid indexes and cardiometabolic biomarkers—anoverview. Nutrients 12(10):3159. 10.3390/nu12103159 10.3390/nu12103159 PMC7602731 33081119

[30] Rahim MA, Uzma M, Al‐Asmari F et al (2025) A comprehensive review on nutritional traits, extraction methods, oxidative stability, encapsulation technologies, food applications and health benefits of omega fatty acids. Food Sci Nutr 13(10):e71008. 10.1002/fsn3.71008 41030839 10.1002/fsn3.71008 PMC12477334

[31] Ntambi JM, Miyazaki M (2004) Regulation of stearoyl-CoA desaturases and role in metabolism. Prog Lipid Res 43(2):91–104. 10.1016/S0163-7827(03)00039-0 14654089 10.1016/s0163-7827(03)00039-0

[32] Khalili TS, Kouřimská L (2022) Assessment of the nutritional quality of plant lipids using atherogenicity and thrombogenicity indices. Nutrients 14(18):3795. 10.3390/nu14183795 36145171 10.3390/nu14183795 PMC9502718

[33] Parzanini C, Parrish CC, Hamel JF et al (2018) Functional diversity and nutritional content in a deep-sea faunal assemblage through total lipid, lipid class, and fatty acid analyses. PLoS One 13(11):e0207395. 10.1371/journal.pone.0207395 30419073 10.1371/journal.pone.0207395 PMC6231680

