# Supplementary material for: Fatty Acids and Protein Content of Underexplored Tropical Palm Fruits
Source: Plant Foods Hum Nutr. 2026 May 15;81(2):63. doi: 10.1007/s11130-026-01521-0 (PMC13179175; doi:10.1007/s11130-026-01521-0)
Supplement: Supplementary file 1 — Supplementary file1 (DOCX 597 KB) [file 11130_2026_1521_MOESM1_ESM.docx]

**Supplementary Material S1. Material and Methods**

**Article: Fatty Acids and Protein Content of Underexplored Tropical Palm Fruits**

**Journal: *Plant Foods for Human Nutrition***

Salima Haddou^1,2^, Mohamed Ezzaitouni^2^, Tarik Chileh-Chelh^2^, Ana Minerva García-Cervantes^2^, Miguel Ángel Rincón-Cervera^2,3^, Ferdaous Al-Ferjani^2^, Ignacio Manuel Rodríguez- García^4^, Chahine Abdelkrim¹, and José Luis Guil-Guerrero^2*^

*jlguil@ual.es

**Material and Methods**

*Samples collection and processing*

The authors manually collected the fruit samples from trees. Species were easily identifiable, as all the trees had the corresponding information sign, and the names of the species were confirmed by the Garden staff. Additionally, published taxonomic literature was used, as well as a comparison with scientific databases. Due to the limited availability of specimens (2–3 trees per species), a stratified sampling design was used to capture intra-tree variability by collecting fruit from three different canopy positions (upper, lower-outer, and inner) with different cardinal orientations, thus ensuring a representative sample of healthy, undamaged fruit. Harvesting was standardized to a single hourly session to minimize diurnal metabolic variations.

Only mature fruits were selected based on external morphological characteristics (e.g., color change, full-size development, and natural detachment from the infructescence). Both fruit pulps and seeds were analysed without having begun dehydration or germination.

The fruits were then manually separated into pulp and seeds. The pulp was carefully removed, and the seeds were cleaned to remove any residual pulp. The stony endocarp (shell) was carefully broken to extract the endosperm, which was the part of the seed analysed.

*Moisture content*

This was determined in pulps and seeds (endosperm) gravimetrically by drying the samples in an oven at 105 °C until constant weight was achieved. Samples were then dried and ground into a fine powder before analysis. Ground samples were stored under controlled conditions until further processing.

*Determination of protein*

Crude protein content in Arecaceae seeds (endoesperm) and pulps was estimated from total nitrogen (N) determined via elemental analysis using a Fisons EA 1108 analyser (Fisons Instruments, USA), applying the standard conversion factor of 6.25.

*Fatty acid analysis*

FA profiles of the fruit organs were determined after direct derivatisation of the lipids into FA methyl esters (FAMEs) as described by Rodríguez-Ruiz et al. [1]. This procedure (also known as one-step extraction/methylation) is a streamlined, rapid analytical technique that allows for the conversion of lipids directly into FA methyl esters (FAMEs) without requiring a separate, time-consuming step for oil extraction [1]. Briefly, 200-500 mg of either fruit pulp or seed (endosperm) were accurately weighed and placed in test tubes, and then 50 μL of the internal standard (nonadecanoic acid, 19:0, Sigma-H3500; Sigma-Aldrich, Barcelona, Spain) in ethanol (10 mg/mL) was added. After that, 2 mL of a methylating mixture (methanol: acetyl chloride, 20:1, v/v) and 1 mL of *n*-hexane were carefully poured over the described material. Tubes were then capped and heated at 100 °C for 30 min. After that, the tubes were cooled to room temperature, 1 mL of distilled water was added to each tube, and the tubes were then centrifuged for 5 min at 2,000 g on a Heraeus Labofuge 200 centrifuge (Thermo Scientific, USA). Subsequently, the hexane layer was collected for GC-FID analysis. FAMEs were analysed in a Focus GC (Thermo Electron, Cambridge, UK), equipped with a flame ionisation detector (FID) and an OmegawaxTM 250 Fused Silica Capillary Column (30 m × 0.25 mm × 0.25 μm film thickness; Supelco, Bellefonte, USA), as previously described [2]. The peak area of the internal standard was used as a reference to calculate the mass of each FA in the resulting chromatograms, and results were reported as FA percentages of total FA. Peaks were identified by retention times obtained for known FAME standards (PUFA No. 1, 47033; from Sigma (Barcelona, Spain). All reagents were purchased from Sigma Chemical (St. Louis, MO).

The quality control for FA analyses was carried out as previously described [3]. The repeatability of the direct methylation was checked by analysing replicates of the same sample daily. The intermediate precision was evaluated by measuring samples on different days throughout the study. Also, blank samples were analysed whenever the methylations were performed. Control oil samples were analysed before and after running samples. For the quality control of GC, a blank sample (hexane) was run along with the samples in every batch. The limits of detection (LOD) and quantification (LOQ) were determined with pure oleic acid (OA, 18:1n-9; purity, 98%) and linoleic acid (LA, 18:2n-6; purity 97.5%), which were diluted in toluene in the 0.001-20 mg/mL range, methylated, and quantified in triplicate by GC-FID. Negative controls were also analysed. The LOD was defined as the minimum concentration at which distinct peaks could be detected above the baseline noise. The estimated LOD for OA and LA were in the range of 0.8-0.9 mg/mL, while LOQ were in the range of 2.4-3.8 mg/mL.

A GC-chromatogram of the FAME of the fruit pulp of *Veitchia metiti* is depicted in Supplementary Figure S1.

**
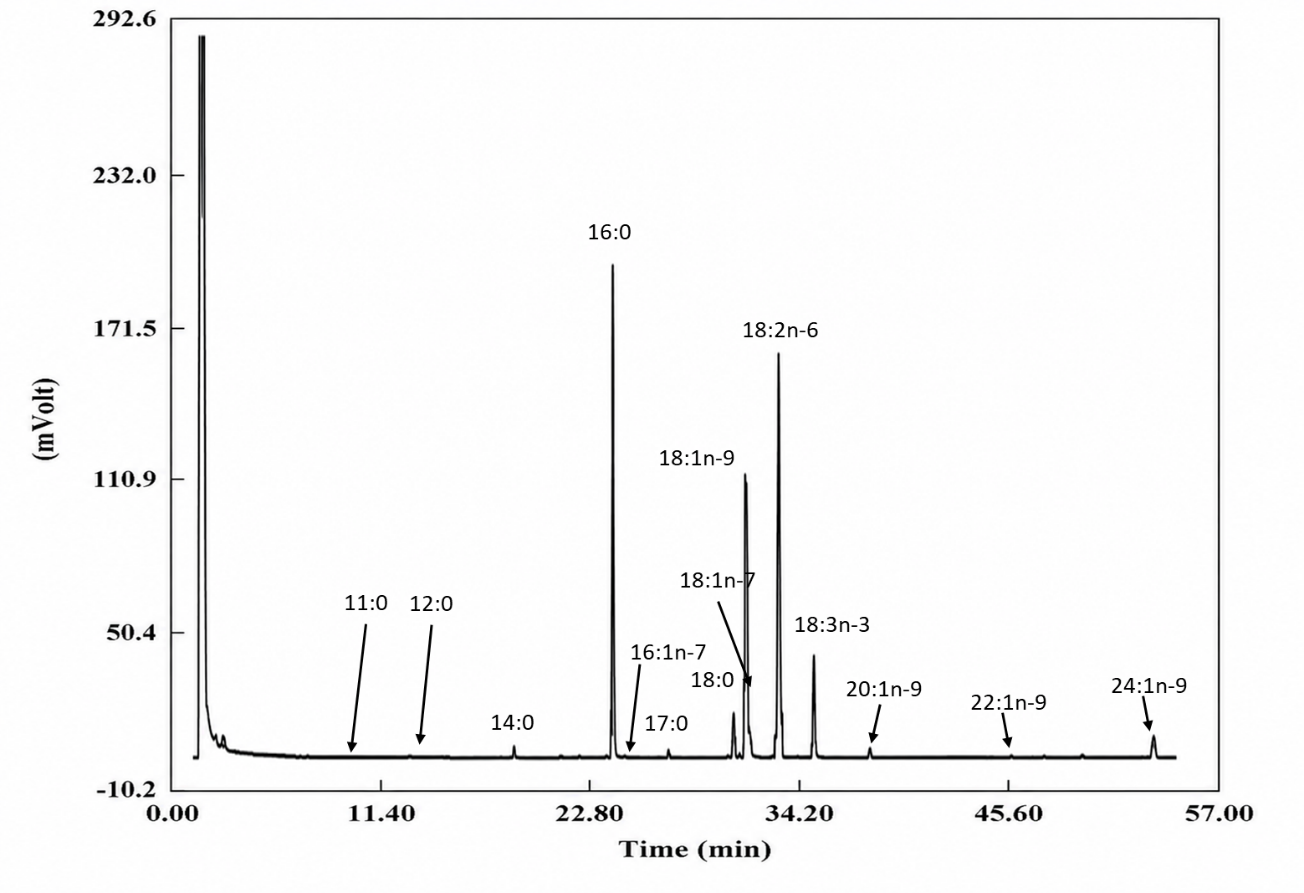
Supplementary Figure S1**. GC-chromatogram of the FAME of the fruit pulp of *Veitchia metiti*

*Stearoyl-CoA desaturase activity indices and nutritional indices*

Stearoyl-CoA desaturase (SCD) activity indices and six FA-based indicators were used to assess nutritional quality: the *n*-6/*n*-3 ratio, PUFA/SFA ratio, atherogenic index (AI), thrombogenic index (TI), and hypocholesterolemic/hypercholesterolemic FA ratio (HH). AI, TI, and HH were calculated according to Chen & Liu [3]. To calculate the nutritional indices, the FA concentrations were expressed as mg/100 g.

**Stearoyl-CoA desaturase (SCD):**

$${SCD}_{i-14}=\left[ \frac{14:1}{14:1+14:0} \right]*100$$

$${SCD}_{i-16}=\left[ \frac{16:1}{16:1+16:0} \right]*100$$

$${SCD}_{i-18}=\left[ \frac{18:1}{18:1+18:0} \right]*100$$

$${SCD}_{i-20}=\left[ \frac{20:1}{20:1+20:0} \right]*100$$

**Atherogenic Index (AI):**

$$AI=\frac{[12:0+\left( 4\times14:0 \right)+16:0]}{\sum Unsaturated FA}$$

**Thrombogenic Index (TI):**

$$TI=\frac{(C14:0+C16:0+C18:0)}{[\left( 0.5\times MUFA \right)+\left( 0.5\times n-6 \right)+\left( 3\times n-3 \right)+(n-3/n-6)]}$$

**Hypocholesterolemic/Hypercholesterolemic FA ratio (HH):**

$$HH=\frac{cis-C18:1+PUFA}{C12:0+C14:0+C16:0}$$

*Statistical analysis*

Statistical analyses were conducted using GraphPad Prism version 9.0 (GraphPad Software, USA). All experiments were conducted by analyzing the pulp and seed of three different fruits collected, each analyzed in triplicate. Data are presented as mean ± standard deviation (SD). Differences between means were evaluated using one-way ANOVA, followed by Tukey’s post hoc test. Statistical significance was set at p < 0.05.

**References**

1. Rodríguez-Ruiz J, Belarbi EH, Sanchez JLG et al (1998) Rapid simultaneous lipid extraction and transesterification for fatty acid analyses. Biotechnol Tech 12(9):689–691. https://doi.org/10.1023/A:1008812904017

2. Lyashenko A, Mercurio F (2019) Looking forward to backward-looking rates: a modeling framework for term rates replacing Libor Available at SSRN 3330240. http://dx.doi.org/10.2139/ssrn.3330240

3. Chen N, Zhou M, Dong X et al (2020) Epidemiological and clinical characteristics of 99 cases of 2019 novel coronavirus pneumonia in Wuhan, China: a descriptive study. The lancet 395(10223):507–513. 10.1016/S0140-6736(20)30211-7
