# Supplementary material for: Fatty Acids and Protein Content of Underexplored Tropical Palm Fruits
Source: Plant Foods Hum Nutr. 2026 May 15;81(2):63. doi: 10.1007/s11130-026-01521-0 (PMC13179175; doi:10.1007/s11130-026-01521-0)
Supplement: Supplementary file 2 — Supplementary file2 (DOCX 28 KB) [file 11130_2026_1521_MOESM2_ESM.docx]

**Supplementary Table S2. Tropical Dates: Usage and Geographic Origin**

**Article: Fatty Acids and Protein Content of Underexplored Tropical Palm Fruits**

**Journal: *Plant Foods for Human Nutrition***

Salima Haddou^1,2^, Mohamed Ezzaitouni^2^, Tarik Chileh-Chelh^2^, Ana Minerva García-Cervantes^2^, Miguel Ángel Rincón-Cervera^2,3^, Ferdaous Al-Ferjani^2^, Ignacio Manuel Rodríguez-García^4^, Chahine Abdelkrim¹, and José Luis Guil-Guerrero^2*^

*jlguil@ual.es

**Supplementary Table S2.** Tropical Palm Species: Usage and Geographic Origin

| Palm species | Method of consumption of fruit/dates | Country of origin | References |
| --- | --- | --- | --- |
| *Syagrus kellyana* | The fruit is edible, but rarely eaten, and sometimes used for oil. | Brazil | [1, 2] |
| *Attalea phalerata* | The fruit is eaten by animals (mammals and birds). It is rarely used for human consumption. | Bolivia, Brazil | [3] |
| *Syagrus orinocensis* | Fruit is edible for animals, but not suitable for human consumption. | Venezuela, Colombia | [4] |
| *Bactris gasipaes* | Fruit is boiled and eaten with salt or processed into flour; used for juices and beer. | Central America, Amazonia | [5] |
| *Veitchia metiti* | Edible fruit. Species grown for ornamental purposes. | Pacific Islands | Although no detailed ethnobotanical documentation was found for *Veitchia metiti*, some general palm-use surveys of the Vanuatu list ([https://vanuatu-data.sprep.org/*Veitchia*](https://vanuatu-data.sprep.org/Veitchia)) indicated their use for palm seeds or endosperm. |
| *Copernicia alba* | Edible fruit consumed by humans; They are used in jams and jellies and are a source of vitamin C. used as livestock feed or for local ecological purposes. | Paraguay, Argentina, Bolivia | [6] |
| *Latania verschaffeltii* | Edible fruit; decorative plant. | Mauritius Island | <https://plantsmarket.in/products> |
| *Wodyetia bifurcata* | Edible fruit consumed by humans, although some sources claim that the flesh of the fruit is technically edible and tasteless | Australia | [7, 8] |

**References**

1. Firmo DHT, Santos SA, Perez MMP et al (2021) Reassessing species boundaries in the Syagrus glaucescens complex (Arecaceae) using leaf anatomy. Botany 99(7):379–387.https://doi.org/10.1139/cjb-2020-0165

2. Baker WJ, Ambal K, Waters et al (2012) Robust absolute magnetometry with organic thin-film devices. Nature Communications 3(1):898 164:190.10.1038/ncomms1895

3. Pott A, Oliveira AK, Damasceno J et al (2011) Plant diversity of the Pantanal wetland. Braz J Biol 71 : 265–273. <https://doi.org/10.1590/S1519-69842011000200005>

4. de Araujo SC, Inae M, Anderson M de Souza et al (2017) The palm tree *Syagrus* oleracea Mart. (Becc.): A review. Sci Hortic 225: 65–73. https://doi.org/10.1016/j.scienta.2017.06.054

5. Clement CR, Santos RP, Desmouliere SJ et al (2009) Ecological adaptation of wild peach palm, its in situ conservation and deforestation-mediated extinction in southern Brazilian Amazonia. PLoS One 4(2): e4564. https://doi.org/10.1371/journal.pone.0004564

6. Bortolotto IM, de Cássia Avellaneda Guimarães R et al (2021) Food composition data: edible plants in Pantanal, in Local food plants of Brazil. Springer 297–324. https://doi.org/10.1007/978-3-030-69139-4

7. Kolakoti Aditya, Prasadarao B, Satyanarayana K et al (2022) Elemental, thermal, and physicochemical investigation of novel biodiesel from *Wodyetia bifurcata* and its properties optimization using artificial neural network (ANN). Automot Exp 5(1) :3–15. https://doi.org/10.31603/ae.6171

8. Mohammed MHH, Mostafa AF (2022) Chemical and biological review on various classes of secondary metabolites and biological activities of Arecaceae (2021-2006). Adv Biomed Pharm Sci 5(3): 113–150.10.21608/jabps.2022.126338.1149
