# Supplementary material for: Fatty Acids and Protein Content of Underexplored Tropical Palm Fruits
Source: Plant Foods Hum Nutr. 2026 May 15;81(2):63. doi: 10.1007/s11130-026-01521-0 (PMC13179175; doi:10.1007/s11130-026-01521-0)
Supplement: Supplementary file 3 — Supplementary file3 (DOCX 20 KB) [file 11130_2026_1521_MOESM3_ESM.docx]

**Supplementary Table S3. Data on Collected Fruits**

**Article: Fatty Acids and Protein Content of Underexplored Tropical Palm Fruits**

**Journal: *Plant Foods for Human Nutrition***

Salima Haddou^1,2^, Mohamed Ezzaitouni^2^, Tarik Chileh-Chelh^2^, Ana Minerva García-Cervantes^2^, Miguel Ángel Rincón-Cervera^2,3^, Ferdaous Al-Ferjani^2^, Ignacio Manuel Rodríguez- García^4^, Chahine Abdelkrim¹, and José Luis Guil-Guerrero^2*^

[*jlguil@ual.es](mailto:*jlguil@ual.es)

**Supplementary Table S3.** Data on collected fruits of the Arecaceae species examined in this study

| Sample Code | Species | Common Name | Native range |
| --- | --- | --- | --- |
| AP | *Attalea phalerata* | Pantanal palm | Southeastern Colombia to Bolivia and Brazil |
| BG | *Bactris gasipaes* | Peach palm | Central and South America |
| CA | *Copernicia alba* | Caranday palm | Southern and western South America |
| LV | *Latania verschaffeltii* | **Yellow latan** | **Rodrigues Island in the Indian Ocean** |
| SK | *Syagrus kellyana* | **Kelly palm** | **Serra do Mar, region of Minas Gerais (Brazil)** |
| SO | *Syagrus orinocensis* | **Orinoco palm** | **Orinoco River basin (Venezuela, Colombia)** |
| VM | *Veitchia metiti* | **Veitchia palm** | **Solomon Islands to SW Pacific** |
| WB | *Wodyetia bifurcata* | **Foxtail palm** | **Cape York Peninsula in the Melville Range of Queensland (Australia)** |
