# Supplementary material for: Fatty Acids and Protein Content of Underexplored Tropical Palm Fruits
Source: Plant Foods Hum Nutr. 2026 May 15;81(2):63. doi: 10.1007/s11130-026-01521-0 (PMC13179175; doi:10.1007/s11130-026-01521-0)
Supplement: Supplementary file 4 — Supplementary file4 (DOCX 26 KB) [file 11130_2026_1521_MOESM4_ESM.docx]

**Supplementary Table S4. Activity indices & nutritional indices**

**Article: Fatty Acids and Protein Content of Underexplored Tropical Palm Fruits**

**Journal: *Plant Foods for Human Nutrition***

Salima Haddou^1,2^, Mohamed Ezzaitouni^2^, Tarik Chileh-Chelh^2^, Ana Minerva García-Cervantes^2^, Miguel Ángel Rincón-Cervera^2,3^, Ferdaous Al-Ferjani^2^, Ignacio Manuel Rodríguez- García^4^, Chahine Abdelkrim¹, and José Luis Guil-Guerrero^2*^

[*jlguil@ual.es](mailto:*jlguil@ual.es)

**Supplementary Table S4.** Activity indices & nutritional indices (fruits & seeds) ^a,b,c,d,e,f^

| Samples | SCD_i___14_ | SCD_i___16_ | SCD_i___18_ | SCD_i___20_ | | AI | TI | HH |
| --- | --- | --- | --- | --- | --- | --- | --- | --- |
| **Palm fruit pulps** | | | | | | | | |
| *A. phalerata* | n.d. | 0.8 ± 0.2^b^ | 64. 2 ± 1.0^e^ | | 59.6 ± 11.8^b^ | 0.6 ± 0.1^de^ | 0.6 ± 0.0^ab^ | 2.2 ± 0.1^b^ |
| *B. gasipaes* | n.d. | 4.0± 0.5^b^ | 84.7 ± 0.0^bc^ | | 50.0 ± 2.6^b^ | 0.4 ± 0.1^e^ | 0.3 ± 0.1^e^ | 1.7 ± 0.0^d^ |
| *C. alba* | n.d. | 26.8 ± 4.0^a^ | 88.9 ± 5.1^ab^ | | 88.7± 16.0^a^ | 1.5 ± 0.1^a^ | 0.6 ± 0.0^bc^ | 0.4 ± 0.1^ef^ |
| *L. verschaffeltii* | n.d. | 1.7 ± 0.3^b^ | 86.3 ± 2.5^b^ | | 17.4± 7.7^d^ | 0.5 ± 0.1^e^ | 0.4 ± 0.0^de^ | 1.3± 0.3^e^ |
| *S. kellyana* | n.d. | 2.3 ± 0.1^b^ | 72.3 ± 0.5^d^ | | 45.7 ± 9.6^bc^ | 0.3 ± 0.1^cd^ | 0.4 ± 0.1^abc^ | 2.0 ± 0.0^c^ |
| *S. orinocensis* | n.d. | 2.6 ± 0.6^b^ | 72.9 ± 0.4^d^ | | 54.3 ± 3.0^b^ | 0.7 ± 0.1^c^ | 0.6 ± 0.1^abc^ | 1.9 ± 0.0^cd^ |
| *V. metiti* | n.d. | 1.8 ± 0.4^b^ | 80.9 ± 1.2^c^ | | 90.6 ± 13.2^a^ | 0.5 ± 0.1^e^ | 0.5 ± 0.0^cd^ | 2.5 ± 0.1^a^ |
| *W. bifurcata* | n.d. | 1.5 ± 0.6^b^ | 92.5 ± 2.7^a^ | | 21.6± 5.9^cd^ | 0.5 ± 0.1^e^ | 0.4 ± 0.1^de^ | 1.4 ± 0.3^e^ |
| *P. dactylifera* var*. Deglet Nour* [1] | n.d. | n.d. | n.d. | | n.d. | 1.4±0.0^a^ | 0.7±0.0^a^ | 0.4±0.1^f^ |
| *P. dactylifera* var.*Medjool* [1] | n.d. | n.d. | n.d. | | n.d. | 1.3±0.0^b^ | 0.7± 0.0^a^ | 0.5±0.1^e^ |
| **Palm fruit seeds** | | | | | | | | |
| *B. gasipaes* | n.d. | n.d. | 71.8 ± 8.6^d^ | | n.d. | 10.6 ± 1.9^a^ | 4.5 ± 0.6^a^ | 0.1 ± 0.0^d^ |
| *C. alba* | n.d. | 35.3 ± 7.6^a^ | 92.9 ± 1.5^ab^ | | n.d. | 2.1 ± 0.2^de^ | 0.4 ± 0.0^d^ | 0.3 ± 0.1^cd^ |
| *L. verschaffeltii* | n.d. | n.d. | 88.8 ± 1.0^abcd^ | | n.d. | 7.3 ± 0.1^b^ | 0.8 ± 0.1^cd^ | 0.1 ± 0.0^d^ |
| *A. phalerata* | n.d. | 3.2 ± 0.1^b^ | 86.9 ± 0.2^bcd^ | | n.d. | 4.1 ± 0.2^c^ | 0.5 ± 0.1^cd^ | 0.1 ± 0.0^d^ |
| *S. orinocensis* | n.d. | n.d. | 81.8 ± 1.9c | | n.d. | 9.4 ± 0.6^a^ | 0.7 ± 0.3^cd^ | 0.1 ± 0.0^d^ |
| *V. metiti* | n.d. | n.d. | 84.3 ± 2.4^cd^ | | n.d. | 3.6 ± 0.0^cd^ | 2.7 ± 0.0^b^ | 0.5 ± 0.2^bc^ |
| *W. bifurcata* | n.d. | n.d. | 90.4 ± 0.3^abc^ | | n.d. | 0.3 ± 0.0^f^ | 0.8 ± 0.1^cd^ | 2.3 ± 0.2^a^ |
| *P. dactylifera* var*. Deglet Nour* [2] | n.d. | n.d. | 93.1 ± 0.3^ab^ | | n.d. | 1.5 ± 0.1^ef^ | 0.9 ± 0.1^c^ | 0.4 ± 0.0^bc^ |
| *P. dactylifera* var.*Medjool* [2] | n.d. | n.d. | 95.0 ± 1.3^a^ | | n.d. | 1.3 ± 0.0^ef^ | 0.8 ± 0.0^cd^ | 0.6 ± 0.0^b^ |

^a^ Data represent means ± standard deviation of indices. ^b^ Differences in values were tested according to one-way ANOVA followed by Tukey’s post hoc test. ^c^ In a row, means followed by different letters are significantly different at p<0.05. Abbreviations: n.d., not detected, SCD: Stearoyl-CoA desaturase activity indices, AI: Atherogenic index, TI: thrombogenic index, and HH: hypocholesterolemic/hypercholesterolemic ratio.

**References**

1. Lahlou A, Chilh-Chelh T, Lyashenko S et al (2022) Arecaceae fruits: Fatty acids, phenolic compounds, and in vitro antitumor activity. Food Biosci 50:102181. <https://doi.org/10.1016/j.fbio.2022.102181>
2. Rincón-Cervera MA, González-Barrio R, Guil-Guerrero JL et al (2023) Arecaceae seeds constitute a healthy source of fatty acids and phenolic compounds. Plants 12(2): 226 <https://doi.org/10.3390/plants12020226>
